# Associação entre Parâmetros Plasmático de Tiol e Níveis de Troponina em Pacientes com Síndrome Coronariana Aguda e Predição de Arritmia Ventricular Hospitalar

**DOI:** 10.36660/abc.20190672

**Published:** 2021-07-07

**Authors:** Mehmet Erdoğan, Selcuk Ozturk, Elçin Özdemir Tutar, Esma Arslan, Muhammet Cihat Çelik, Serdal Baştuğ, Salim Neşelioğlu

**Affiliations:** 1 Ministry of Health Ankara City Hospital-Cardiology Ankara Turquia Ministry of Health Ankara City Hospital-Cardiology, Ankara - Turquia; 2 Yozgat Bozok University Faculty of medicine-Cardiology Yozgat Turquia Yozgat Bozok University Faculty of medicine-Cardiology, Yozgat - Turquia; 3 Yildirim Beyazit University Faculty of Medicine-Biochemistry Ankara Turquia Yildirim Beyazit University Faculty of Medicine-Biochemistry, Ankara - Turquia; 4 Yildirim Beyazit University Faculty of Medicine-Biochemistry Ankara Turquia Yildirim Beyazit University Faculty of Medicine-Biochemistry, Ankara - Turquia

**Keywords:** Aterosclerose, Doença da Artéria Coronariana, Síndrome Coronariana Aguda, Estresse Oxidativo, Arritmias Cardíacas, Acetil-CoAc-Aciltransferase

## Abstract

**Fundamento:**

As arritmias ventriculares (AVs) são a principal causa de mortalidade e morbidade hospitalar em pacientes com síndrome coronariana aguda (SCA) e sua relação com o tiol é desconhecida.

**Objetivo:**

Investigar a relação entre os níveis plasmáticos de tióis e os níveis de troponina em pacientes com SCA e estimar o desenvolvimento de AV intra-hospitalar durante a internação.

**Método:**

O estudo incluiu 231 pacientes consecutivos com SCA com supradesnivelamento do segmento ST (SCA-SDST) e pacientes com SCA sem supradesnivelamento do segmento ST (SCA-SSDST). Após a aplicação dos critérios de exclusão, 191 pacientes foram incluídos na análise estatística. Os pacientes foram classificados em dois grupos: grupo SCA-SDST (n=94) e grupo SCA-SSDST (n=97). Os níveis plasmáticos de tiol, dissulfeto e troponina foram medidos e a razão de troponina para tiol nativo (RTTN) foi calculada. Considerou-se estatisticamente significativo um valor de p bilateral inferior a 0,05.

**Resultados:**

Tiol nativo plasmático, tiol total, dissulfeto e suas razões foram semelhantes entre os grupos. A RTTN se mostrou significativamente maior no grupo SCA-SDST em comparação com o grupo SCA-SSDST. Houve correlação negativa significativa entre os níveis de troponina e tiol. Verificou-se que o tiol nativo é preditor independente do desenvolvimento de AV em pacientes com SCA-SDST e em todos os pacientes com SCA. Verificou-se que o RTTN é preditor independente do desenvolvimento de AV em pacientes com SCA-SSDST e em todos os pacientes com SCA.

**Conclusão:**

Os níveis plasmáticos de tiol podem ser usados para identificar pacientes com alto risco de desenvolvimento de AV intra-hospitalar em pacientes com SCA. A correlação entre os níveis de troponina e tiol pode sugerir que os tióis possam ser marcadores importantes para o diagnóstico e prognóstico da SCA com a ajuda de estudos futuros.

## Introdução

A aterosclerose é uma doença multifatorial causada por dano endotelial em pessoas com fatores de risco como dislipidemia, diabetes, tabagismo e predisposição genética. A síndrome coronariana aguda (SCA), resultado dessa doença, inclui pacientes com supradesnivelamento persistente do segmento ST (SCA-SDST) e pacientes sem supradesnivelamento persistente do segmento ST (SCA-SSDST) detectada por eletrocardiografia (ECG).^1^ O supradesnivelamento do segmento ST está principalmente associado à oclusão coronária aguda total, enquanto a SCA-SSDST está principalmente relacionada à placa aterosclerótica sensível e trombose subtotal.

Nos últimos dez anos, diversos estudos demonstraram o papel da superprodução de espécies reativas de oxigênio (ERO) e o estresse oxidativo resultante na patogênese da aterosclerose.^2^ De fato, o processo metabólico do ERO desempenha papeis significativos nas funções fisiológicas das células e esse processo é contrabalançado pelo sistema antioxidante do organismo. A oxidação de partículas de lipoproteína de baixa densidade (LDL) consiste na fase preliminar e principal da aterosclerose. Além disso, a formação de ERO contribui para os processos de apoptose, inflamação e proliferação celular.^3^

O status da homeostase de tiol e dissulfeto exerce um papel importante na proteção antioxidante, desintoxicação, apoptose e regulação da atividade enzimática.^4^ Estudos recentes mostraram que alterações no nível plasmático de tiol e o equilíbrio de dissulfeto estão associados a diversas doenças cardiovasculares, como angina pectoris estável,^5^ agudo infarto do miocárdio (IAM),^6^ síndrome cardíaca X,^7^ fluxo lento coronariano,^8^ hipertensão primária^9^ e diabetes mellitus (DM).^10^ Sabe-se que as arritmias ventriculares (AVs), que podem ser decorrentes de necrose miocárdica, desequilíbrio autonômico e eletrolítico, acidose e lesão de reperfusão são as principais causas de mortalidade e morbidade intra-hospitalar em pacientes com SCA.^11^ No entanto, não há estudos na literatura sobre a relação entre processo oxidativo, necrose miocárdica e desenvolvimento de AV intra-hospitalar em pacientes com SCA. Nesse contexto, nosso objetivo foi investigar a relação entre os níveis plasmáticos de tiol e troponina em pacientes com SCA e sua associação com o desenvolvimento de AV durante o seguimento hospitalar.

## Métodos

Este estudo transversal observacional foi conduzido de fevereiro a maio de 2018 e incluiu 231 pacientes consecutivos de nossa clínica de cardiologia. Pacientes internados em unidade de terapia intensiva coronariana com diagnóstico de SCA submetidos a angiocoronariografia foram incluídos no estudo de forma prospectiva. Pacientes com doenças infecciosas ou inflamatórias ativas, malignidade, distúrbios hematológicos, doença renal importante, doença hepática importante, doença reumática, doença valvar importante, pacientes em uso de antioxidantes e/ou em terapia de reposição de vitaminas e pacientes em choque cardiogênico foram excluídos do estudo. Além disso, pacientes com dados clínicos insuficientes também foram excluídos. O protocolo do estudo foi aprovado pelo comitê de ética local (Nº: 39/Data: 21.02.2018) e foi conduzido de acordo com os princípios éticos da Declaração de Helsinque. Todos os pacientes assinaram o consentimento informado.

Após a aplicação dos critérios de exclusão, 191 pacientes foram incluídos na análise estatística. Os pacientes foram classificados em dois grupos: Grupo SCA-SDST (n=94) e grupo SCA-SSDST (n=97). Foi feito diagnóstico de SCA de acordo com as diretrizes atuais por meio de anamnese, ECG, métodos de imagem e níveis de troponina. Foram avaliados os parâmetros clínicos basais dos pacientes. Mortalidade intra-hospitalar, ocorrência de AV e tempo de internação também foram avaliados e registrados. O diagnóstico de SCA-SDST incluiu pacientes com dor torácica de início agudo e supradesnivelamento persistente do segmento ST ao ECG.^12^ O diagnóstico de SCA-SSDST incluiu pacientes com dor torácica de início agudo, sem supradesnivelamento persistente do segmento ST ao ECG e/ou níveis de troponina cardíaca maiores que o limite superior da faixa normal.^13^ Definiu-se hipertensão arterial como pacientes com medidas repetidas de pressão arterial ≥140/90 mm Hg ou diagnóstico prévio de hipertensão com medicamentos anti-hipertensivos. Definiu-se DM como níveis de glicose plasmática em jejum acima de 126 mg/dL em múltiplas medições ou nível de glicose acima de 200 mg/dL em qualquer medição ou uso ativo de medicamentos antidiabéticos e/ou terapia com insulina. Definiu-se tabagismo como tabagismo atual nos últimos seis meses. Definiu-se hipercolesterolemia como nível basal de colesterol >200 mg/dl e/ou nível de colesterol LDL >130 mg/dl ou hipercolesterolemia previamente diagnosticada e tratada.

Definiu-se AV intra-hospitalar como taquicardia ventricular (TV) sustentada ou não, e fibrilação ventricular (FV). Determinou-se o diagnóstico a partir de registros de ECG e/ou telemetria no início do estudo, de acordo com o estado clínico dos pacientes durante a internação. Além disso, os registros de telemetria foram analisados retrospectivamente no computador em busca de outros ataques de AV antes da alta hospitalar. Extrassístole ventriculares com mais de três batimentos consecutivos foram definidas como TV. TVs com duração superior a trinta segundos foram definidas como sustentados, e as com duração mais curta foram definidas como TV não sustentada.

Para pacientes com SCA-SDST, calculou-se o escore de risco de Trombólise em Infarto do Miocárdio (TIMI), composto por 8 itens, que prevê mortalidade por todas as causas em 30 dias. Para pacientes com SCA-SSDST, calculou-se o escore de risco TIMI composto por 7 itens, que prevê mortalidade por todas as causas, IM novo ou recorrente, ou isquemia recorrente importante exigindo revascularização urgente em 14 dias.^14^ Em todos os pacientes, calculou-se o escore de risco do Registro Global de Eventos Coronários Agudos (GRACE) para estimar a probabilidade de óbito intra-hospitalar.^15^

Realizou-se ecocardiografia transtorácica com transdutor de arranjo faseado 3S–RS 1,5–3,6 MHz com aparelho de ultrassom General Electric Vivid 7 (GEMS Ultrasound, Israel) nas primeiras 48 horas de hospitalização. Calculou-se a fração de ejeção ventricular esquerda (FEVE) a partir das imagens apicais 2 e 4 câmaras pelo método de Simpson modificado. Realizou-se angiocoronariografia por meio do aparelho Siemens Axiom Sensis XP por canulização da artéria femoral aplicando-se a técnica padrão de Judkins. Além do ácido acetilsalicílico, todos os pacientes receberam terapia antiplaquetária dupla com clopidogrel, prasugrel ou ticagrelor de acordo com seu estado clínico.

As medições das amostras de sangue venoso dos pacientes foram feitas na admissão. Estudos bioquímicos de rotina foram realizados no autoanalisador Hitachi 747. Mediu-se o colesterol da lipoproteína de alta densidade (HDL) após a precipitação de sulfato de dextrano e magnésio. Calculou-se o colesterol LDL pelo método de Friedewald. Colesterol, triglicerídeos em jejum e concentrações plasmáticas de colesterol HDL foram medidos pelo método de limpeza química enzimática no analisador Cobas 6000 (Roche Diagnostics GmbH, Mannheim, Alemanha). Os níveis séricos de troponina I foram medidos quantitativamente no analisador Elecsys 2010 (Roche Diagnostics, Basel, Suíça). Os níveis séricos de enzimas cardíacas (CK, CK-MB e troponina I) foram medidos na internação, e repetidos todos os dias. Os níveis plasmáticos de tiol e dissulfeto foram medidos de acordo com o método desenvolvido por Erel e cols. imediatamente após a internação. Amostras sanguíneas foram coletadas em tubos contendo ácido etilenodiaminotetraacético (EDTA) para determinar os níveis de tiol e dissulfeto. Resumidamente, as ligações de dissulfeto degradável foram reduzidas para formar grupos tiol funcionais livres. Posteriormente, utilizou-se redutor boro-hidreto de sódio não utilizado, tendo sido extraído com formaldeído e, em seguida, todos os grupos de tiol nativo e reduzido foram determinados após reação com ácido 5,5-ditiobis- (ácido 2-nitrobenzoico). Os níveis de dissulfeto dinâmico foram calculados pela metade da diferença entre o tiol total e o nativo. Após a determinação dos níveis de tiol nativo e dissulfeto, calculou-se a razão tiol nativo-dissulfeto. Usando este método, determinou-se a homeostase dinâmica da razão tiol-dissulfeto de forma mais fácil e econômica em aproximadamente dez minutos.^16^

## Análise estatística

Todas as análises foram realizadas no equipamento IBM SPSS Statistics for Macintosh, versão 24.0 (IBM Corp., Armonk, Nova York, EUA). Utilizou-se o teste de Kolmogorov-Smirnov de amostra única para avaliar a distribuição das variáveis numéricas. De acordo com os resultados desse teste, aplicou-se o teste T de duas amostras independentes aos dados numéricos em conformidade com a distribuição normal e os resultados foram inseridos como média e desvio padrão. Por outro lado, utilizou-se o teste U de Mann-Whitney para variáveis distribuídas assimetricamente. Considerando os resultados desse teste, foram utilizados os valores medianos e do intervalo interquartil. Utilizou-se o teste qui-quadrado para as variáveis categóricas. Aplicou-se o teste exato de Fisher nos casos em que o teste do qui-quadrado não pôde ser aplicado. Para análises de correlação, utilizou-se a análise de correlação de Pearson para dados com distribuição normal. Ou então, utilizou-se a análise de correlação de Spearman. Os preditores independentes de AV intra-hospitalar foram determinados por meio de análise de regressão logística. Variáveis que pudessem ter uma relação clínica com AV intra-hospitalar, como LDL, idade, FEVE, níveis séricos de potássio e magnésio, razão neutrófilos-linfócitos (RNL), tempo até a internação, razão troponina/tiol nativo (RTTN) e troponina e tiol nativo foram incluídos na análise de regressão logística. Para evitar a multicolinearidade, realizamos uma análise multivariada usando três modelos separadamente. Cada modelo multivariado incluiu apenas um marcador (RTTN, troponina, tiol nativo). Realizou-se análise de regressão logística separadamente para todos os grupos com SCA-SDST, SCA-SSDST e todos os pacientes com ACS. Utilizou-se a análise da curva *receiver operating characteristic* (ROC) foi usada para determinar os valores de corte (índice de Youden^17^ ) para a sensibilidade e especificidade da RTTN na previsão de AV. Considerou-se estatisticamente significativo um valor de *p* bilateral inferior a 0,05.

## Resultados

A Tabela 1 apresenta as características demográficas, os parâmetros laboratoriais e as características clínicas dos grupos de pacientes. Não houve diferença entre os grupos em termos de idade, sexo, índice de massa corporal e fibrilação atrial. A porcentagem de hipertensão, DM e o tempo até a internação foi maior no grupo SCA-SSDST, enquanto a porcentagem de fumantes foi maior no grupo SCA-SDST. As contagens de neutrófilos, plaquetas e RNL foram significativamente maiores no grupo SCA-SDST em comparação com o grupo SCA-SSDST. Contagens de linfócitos, hemoglobina, albumina, eletrólitos séricos, incluindo potássio, cálcio e magnésio, testes de função renal e hepática e parâmetros lipídicos, exceto triglicerídeos, foram comparáveis entre os grupos. Os valores máximos das enzimas cardíacas, incluindo CK-MB e troponina, foram significativamente maiores no grupo SCA-SDST do que no grupo SCA-SSDST, enquanto a FEVE se mostrou menor no grupo SCA-SDST. Os níveis de tiol nativo plasmático, tiol total, dissulfeto, razão dissulfeto-tiol nativo, razão dissulfeto-tiol total e razão tiol nativo-tiol total foram semelhantes entre os grupos. No entanto, a RTTN foi significativamente maior no grupo SCA-SDST em comparação com o grupo SCA-SSDST. Além disso, o escore GRACE foi significativamente maior no grupo SCA-SDST em comparação com o grupo SCA-SSDST. No total, 23 pacientes desenvolveram AV hospitalar. Dos pacientes que desenvolveram AV hospitalar, 14 pacientes tiveram AV sustentada, enquanto 9 pacientes tiveram AV não sustentada. Não houve diferença entre os grupos quanto à ocorrência de AV, tempo de internação e mortalidade intra-hospitalar.

**Tabela 1:** – Características demográficas e achados laboratoriais da população do estudo

Variáveis	SCA-SDST (n=94)	SCA-SSDST (n=97)	Valor de p
Idade, anos	58.7±11,1	62.1±13,7	0,06
Sexo masculino, n (%)	80 (85)	71 (73)	0,05
IMC (kg/m^2^)	26.9±6,9	28.5±5,6	0,14
Hipertensão, n (%)	39 (41)	61 (62)	0,003*
Diabetes mellitus, n (%)	22 (23)	43 (44)	0,02*
Tabagismo, n (%)	65 (69)	44 (45)	0,001*
Fibrilação atrial, n (%)	0 (0)	8 (8)	0,07
Tempo até a internação, horas	2 (1–5)	28 (7–72)	<0,001*
Contagem de neutrófilos (K/uL)	7650 (5500–11525)	6100 (4600–8150)	<0,001*
Contagem de linfócitos (K/uL)	1900 (1222–3025)	1900 (1525–2500)	0,81
RNL	5,0 (2,0–7,9)	2,7 (2,1–4,5)	0,02*
Contagem de plaquetas (K/uL)	252±79	231±63	0,04*
Hemoglobina (g/dl)	14.4±1,7	13.7±1,8	0,08
Albumina (g/dl)	4.1±0,4	4.2±0,3	0,21
Colesterol total (mg/dl)	182±42	194±53	0,07
HDL (mg/dL)	37 (31–45)	39 (33–47)	0,06
LDL (mg/dl)	119±33,5	116±36,5	0,57
Triglicerídeos (mg/dl)	97,5 (60–154)	130 (91–189)	<0,001*
Potássio (mmol/L)	4.3±0,4	4.4±0,5	0,14
Creatinina (mg/dl)	0,9 (0,7–1,0)	0,9 (0,7–1,1)	0,31
TFG (ml/min/m^2^)	90 (72–144)	86 (60–96)	0,07
Cálcio (mg/dl)	9.2±0,6	9.3±0,5	0,17
Magnésio (mg/dl)	2,0 (1,9–2,2)	2,0 (1,9–2,2)	0,79
AST (U/L)	30,5 (20–52)	30 (21–39)	0,34
ALT (U/L)	24 (16–34)	21 (14–30)	0,13
Massa CK-MB (ng/mL)	100 (26–209)	13 (3–66)	<0,001*
Troponina I (ug/L)	2571 (736–4810)	365 (43–1394)	<0,001*
FEVE (%)	43.9±8,5	49±11,2	<0,001*
Troponina/tiol nativo	6,7 (1,9 -15,8)	1,0 (0,1 - 3,8)	<0,001*
Tiol plasmático nativo (µ mol/l)	359.8±78,4	366.6±68,5	0,52
Tiol plasmático total (µ mol/l)	401.3±81,7	407.6±74,4	0,57
Dissulfeto (µmol/l)	20.1±10,8	19.0±11,9	0,53
Dissulfeto/tiol nativo	0,04 (0,03–0,08)	0,05 (0,03–0,07)	0,49
Dissulfeto/tiol total	0,04 (0,03–0,07)	0,04 (0,02–0,06)	0,53
Tiol nativo/tiol total	0,90 (0,86–0,94)	0,91 (0,87–0,95)	0,16
Escore de risco TIMI	2 (1–4)	4 (3–5)	-
Escore GRACE	151.7±26,6	133.7±35,1	<0,001*
Arritmia ventricular, n (%)	13 (13,8)	10 (10,3)	0,45
Período de internação, dias	4 (3–5)	4 (3–5)	0,26
Mortalidade, n (%)	3 (3)	1 (1)	0,36

ALT: alanina transaminase; AST: aspartato transaminase; IMC: índice de massa corporal; CK-MB: creatina quinase-MB; TFG: taxa de filtração glomerular; HDL: lipoproteína de alta densidade; IIQ: intervalo interquartil; LDL: lipoproteína de baixa densidade; FEVE: fração de ejeção ventricular esquerda; RNL: razão neutrófilos/linfócitos; SCA-SSDST: síndrome coronariana aguda sem supradesnivelamento do segmento ST; desvio padrão; SST: supradesnivelamento do segmento ST. Os parâmetros foram expressos como média±DP e mediana [IIQ]. *Considerou-se significativo para análises estatísticas um p<0,05.

**Figura 1:**
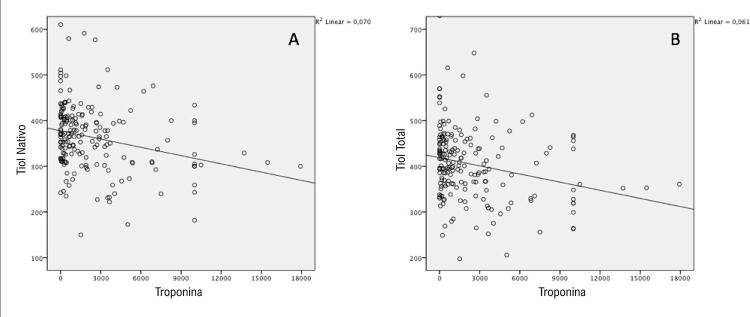
– *Relação entre troponina e níveis de tiol nativo e total.*

A análise de correlação de variáveis múltiplas com tiol nativo, tiol total e RTTN em toda a população do estudo é apresentada na Tabela 2 . Os níveis de tiol nativo apresentaram correlação significativa negativa com idade, escore GRACE, RNL, internação hospitalar e troponina ( Figura 1A ), e correlação significativa e positiva com TFG, FEVE e albumina. Os níveis de tiol total apresentaram correlação significativa e negativa com idade, escore GRACE, tempo de internação hospitalar e troponina ( Figura 1B ), e correlação significativa positiva com TFG, FEVE e albumina. Os níveis de RTTN apresentaram correlação inversa significativa com a FEVE, e correlação positiva significativa com o escore GRACE, RNL, CKMB e tempo de internação hospitalar. O coeficiente de correlação foi fraco ou moderado para todas as variáveis significativas incluídas na análise.

**Tabela 2:** – Parâmetros de índice de tiol plasmático nativo, tiol total e troponina/tiol nativo em pacientes com síndrome coronariana aguda e sua correlação com as variáveis

Variáveis	Tiol nativo		Tiol total		Razão troponina-tiol nativo	

	r	p	r	p	r	p
Idade	-0,25	<0,001*	-0,26	<0,001*	+0,06	0,34
TFG	+0,33	<0,001*	+0,33	<0,001*	-0,13	0,06
Escore GRACE	-0,36	<0,001*	-0,35	<0,001*	+0,40	<0,001*
FEVE (%)	+0,19	0,009*	+0,18	0,01*	-0,42	<0,001*
RNL	-0,14	0,04*	-0,14	0,054	+0,34	<0,001*
Albumina	+0,39	<0,001*	+0,41	<0,001*	-0,12	0,07
Troponina	-0,27	<0,001*	-0,28	<0,001*	-	-
Massa CK-MB	-0,11	0,12	-0,10	0,15	+0,63	<0,001*
Período de internação	-0,22	0,002*	-0,16	0,02*	+0,23	0,001*
LDL	+0,10	0,14	+0,13	0,06	+0,04	0,57

CK-MB: creatina quinase-MB; TFG: taxa de filtração glomerular; LDL: lipoproteína de baixa densidade; FEVE: fração de ejeção ventricular esquerda; RNL: razão neutrófilo-linfócito. *Considerou-se significativo para análises estatísticas um p<0,05.

**Figura 2:**
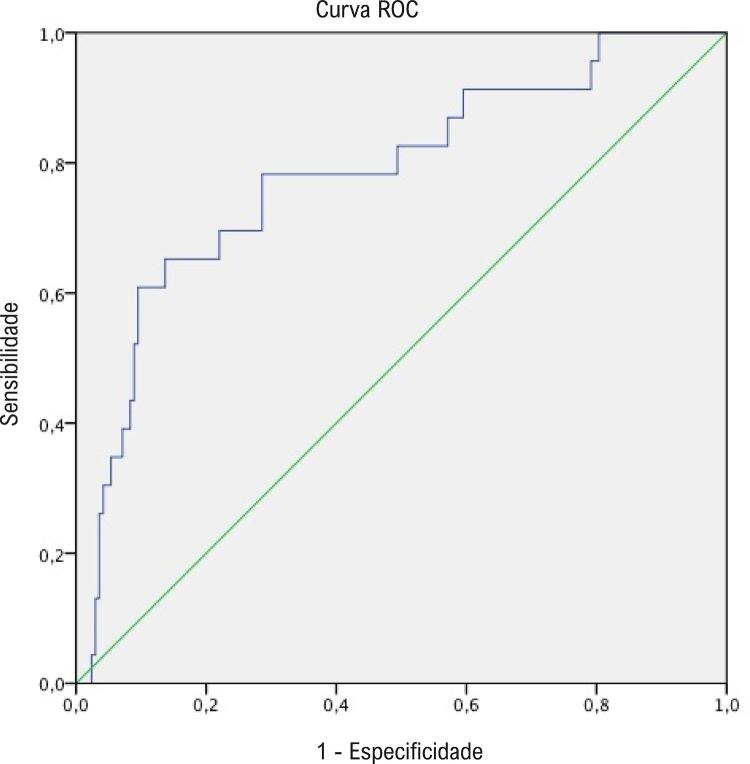
– *Análises da curva receiver operating characteristic (ROC) para predição do desenvolvimento de arritmia ventricular em todos os pacientes.*

A Tabela 3 mostra a análise de regressão logística dos preditores de AV intra-hospitalar para pacientes com SCA-SDST, pacientes com SCA-SSDST e todos os pacientes com SCA. Considerou-se o tiol nativo um preditor independente do desenvolvimento de AV em pacientes com supradesnivelamento do segmento ST, ao passo que a RTTN foi considerada preditora independente do desenvolvimento de AV em pacientes com SCA-SSDST. Quando se trata de toda a população de pacientes com ACS, verificou-se que a RTTN e o tiol nativo são preditores independentes do desenvolvimento de AV. Em todos os pacientes com ACS, a área sob a curva para RTTN foi de 0,783 e o valor de corte da RTTN (6,13) esteve associado a 78% de sensibilidade e 72% de especificidade na predição de AV intra-hospitalar, conforme mostrado na Figura 2 .

**Tabela 3:** – Preditores independentes de arritmia ventricular por análise de regressão logística

Variáveis	OR	IC 95%		valor de p

		Inferior	Superior	
**Arritmia ventricular/SDST**				
LDL	1,021	0,997	1,045	0,08
Idade	1,026	0,965	1,091	0,40
FEVE (%)	1,044	0,943	1,155	0,40
Potássio	0,849	0,214	3,366	0,81
Magnésio	1,072	0,121	9,518	0,95
RNL	1,142	0,997	1,308	0,05
Tempo até a internação	1,016	0,994	1,038	0,15
Troponina/tiol nativo	1,030	0,981	1,081	0,23
Troponina	1,000	1,000	1,000	0,64
Tiol nativo	0,962	0,937	0,986	0,03*
**Nagelkerke R** ^**2**^ **= 0,257, p: 0,07**				
**Arritmia ventricular/SCA-SSDST**				
LDL	0,970	0,940	1,001	0,05
Idade	1,086	0,985	1,196	0,09
FEVE (%)	1,034	0,917	1,165	0,58
Potássio	1,278	0,218	7,488	0,78
Magnésio	0,105	0,001	11,00	0,34
RNL	1,102	0,919	1,321	0,29
Tempo até a internação	1,003	0,988	1,019	0,68
Troponina/tiol nativo	1,263	1,061	1,504	0,009*
Troponina	1,001	1,000	1,001	0,05
Tiol nativo	0,969	0,947	0,992	0,08
**Nagelkerke R** ^**2**^ **= 0,592, p<0,001**				
**Arritmia ventricular/Todos os pacientes**				
LDL	1,001	0,987	1,015	0,90
Idade	1,040	0,998	1,084	0,06
FEVE (%)	0,994	0,940	1,051	0,82
Potássio	0,867	0,346	2,169	0,76
Magnésio	0,681	0,096	4,845	0,70
RNL	1,087	1,000	1,182	0,05
Tempo até a internação	1,002	0,995	1,008	0,57
Troponina/tiol nativo	1,059	1,022	1,098	0,002*
Troponina	1,000	1,000	1,000	0,10
Tiol nativo	0,976	0,966	0,986	<0,001*
**Nagelkerke R** ^**2**^ **=0,240, p: 0,001**				

Análise de regressão logística multivariada. O modelo de regressão incluiu idade, lipoproteína de baixa densidade (LDL), fração de ejeção ventricular esquerda (FEVE, %), potássio sérico, magnésio sérico, razão neutrófilos-linfócitos (RNL) e tempo até a internação hospitalar como possíveis variáveis independentes. IC 95%: intervalo de confiança de 95%; OR: razão de chances. *Considerou-se significativo para análises estatísticas um p<0,05.

## Discussão

Os achados do presente estudo demonstraram uma correlação negativa entre os níveis de troponina e tiol plasmático em pacientes com SCA. Além disso, principalmente em pacientes com SCA-SSDST e em toda a população de pacientes com SCA, a RTTN se mostrou um marcador forte e independente para a predição de AV intra-hospitalar. Até onde sabemos, este é o primeiro estudo na literatura que demonstra a relação entre o tiol e o desenvolvimento de AV intra-hospitalar em pacientes com SCA.

Um dos principais mecanismos fisiopatológicos da aterosclerose é o aumento do estresse oxidativo com inflamação.^18^ O nível sérico de tiol, que é um indicador de estresse oxidativo, pode ser detectado manualmente por eletroforese capilar fluorescente e/ou sistemas luminescentes. No entanto, esses sistemas são caros, demorados e difíceis de aplicar. Um método colorimétrico desenvolvido por Erel et al. em 2014 levou à determinação automática dos níveis de tiol. Além disso, esse novo método é mais preciso, rápido e barato em comparação com os métodos anteriores.^16^ Por outro lado, a RNL, que é um marcador inflamatório de fácil acesso e barato, é um parâmetro que demonstra resposta inflamatória sistêmica em diversas doenças cardiovasculares e não cardiovasculares.^1^ Em um estudo realizado por Altiparmak et al.,^7^ os níveis de tiol sérico total e nativo mostraram-se menores em pacientes com síndrome cardíaca X e os níveis totais de tiol sérico e nativo mostraram-se negativamente correlacionados com a RNL.^7^ Em nosso estudo, os níveis de RNL mostraram-se significativamente maiores no grupo de pacientes SCA-SDST em comparação com o grupo de pacientes SCA-SSDST e houve uma correlação negativa entre a RNL e os níveis de tiol em toda a população com SCA. Com base nesses achados, pode-se especular que o tiol plasmático, um marcador de proteção antioxidante no corpo humano, está negativamente associado ao aumento da inflamação em pacientes com SCA.

O estresse oxidativo inicia o desenvolvimento da aterosclerose por meio da peroxidação lipídica e formação de radicais livres nos primeiros estágios e por meio da inflamação vascular em estágios avançados.^19^ A instabilidade da placa aterosclerótica é desencadeada pelo aumento do estresse oxidativo e radicais reativos de oxigênio, enquanto a capacidade antioxidante diminui durante esse processo. Posteriormente, o equilíbrio do sistema oxidativo e antioxidante, que é um importante regulador da proteção celular, desintoxicação, apoptose e atividades enzimáticas, começa a se deteriorar.^20^ Um estudo anterior mostrou que os níveis de marcadores de estresse oxidativo aumentam após o IAM e reperfusão.^21^ Em nosso estudo, houve uma correlação negativa e estatisticamente significativa entre os níveis plasmáticos de tiol e a idade e a troponina, ao passo que houve uma correlação positiva e estatisticamente significativa entre os níveis de tiol e a FEVE. Esses resultados correspondem aos de um estudo publicado por Kundi et al.^6^ Sivri et al. demonstraram menores níveis de tiol total plasmático e tiol nativo em pacientes com SCA-SSDST em comparação com os pacientes do grupo controle. Além disso, eles demonstraram uma relação inversa entre o escore GRACE e os níveis totais de tiol plasmático e tiol nativo.^22^ De acordo com nosso estudo, que incluiu pacientes SCA-SDST e SCA-SSDST, observou-se uma correlação negativa entre o escore GRACE e os parâmetros de tiol. A correlação do tiol com esses fortes marcadores diagnósticos e prognósticos, como a troponina e o escore GRACE, pode reforçar que o tiol pode ser um marcador importante para o diagnóstico e prognóstico de SCA com a ajuda de estudos futuros.

Mais de 80% das mortes súbitas cardíacas se devem a doença arterial coronariana aterosclerótica (DAC), e a causa mais comum são as AVs devido à isquemia resultante de DAC e SCA.^23^ Portanto, a predição precoce, o diagnóstico e o tratamento eficaz das AVs são muito importantes. Durante o estresse oxidativo, aumenta a sensibilidade do coração de idosos à FV. Em um estudo, o estresse oxidativo induzido por peróxido de hidrogênio apresentou associação ao aumento de pós-potenciais precoces e atividade deflagrada em miócitos ventriculares.^24^ Embora o miocárdio tenha um forte mecanismo de defesa, é suscetível ao estresse oxidativo devido à sua alta carga de trabalho e demanda por oxigênio. Como resultado da isquemia, ocorre diminuição dos níveis de antioxidantes, como a superóxido dismutase mitocondrial e a glutationa intracelular contra os radicais livres de oxigênio. Além disso, a produção de radicais livres de oxigênio aumenta nas mitocôndrias e leucócitos, e a produção de metabólitos de oxigênio aumenta durante o período de reperfusão, que podem ser mais tóxicos pela reintrodução de oxigênio. O estresse oxidativo causa primeiramente a oxidação dos grupos tiol, levando a um dano reversível no estágio inicial. Em estágios posteriores, esse processo leva à necrose acelerada e à predisposição a arritmias. No período inicial, pode induzir desequilíbrio elétrico da membrana miocárdica, alterações na permeabilidade dos canais iônicos e indução de alterações arritmogênicas no padrão potencial de ação ventricular.^21^ Observou-se aumento na duração do potencial de ação após a exposição aos radicais livres de oxigênio seguido do aparecimento de pós-potenciais precoces e pós-potenciais tardios.^25 , 26^ Com base em relatórios publicados anteriormente, formulamos a hipótese de que os níveis de tiol podem predizer o desenvolvimento de AV em pacientes com SCA e verificamos que a RTTN pode ser usada para predizer o desenvolvimento de AV em pacientes com SCA-SSDST, mas não em pacientes com SCA-SDST. Essa discordância pode ter origem nas diversidades fisiopatológicas e clínicas entre SCA-SDST e SCA-SSDST. A SCA-SDST é resultado da oclusão total do vaso com coágulo rico em fibrina, ao passo que a SCA-SSDST é resultado da oclusão subtotal do vaso com coágulo rico em plaquetas. Além disso, o tempo até a internação hospitalar é menor em pacientes com SCA-SDST e, consequentemente, o tempo de revascularização é mais rápido em comparação com pacientes com SCA-SSDST. Portanto, pode-se especular que não há tempo suficiente para o processo oxidativo progredir para o plasma em nível celular em pacientes com SCA-SDST. No entanto, devido ao fato de que o tempo de revascularização é maior em pacientes com SCA-SSDST, a progressão do estresse oxidativo dos cardiomiócitos para o plasma pode ser mais pronunciada. Além disso, os pacientes com SCA-SSDST têm mais idade do que os pacientes com SCA-SDST e sofrem de comorbidades como DM e/ou hipertensão com mais frequência em comparação com pacientes com SCA-SDST, conforme mostrado em nosso grupo de estudo. Essas comorbidades podem ter afetado o estado oxidativo em pacientes com SCA-SSDST.

Nosso estudo apresenta diversas limitações. Em primeiro lugar, o número de amostras é relativamente pequeno e este é um estudo unicêntrico. Em segundo lugar, este é um estudo observacional e deve ser respaldado por novos estudos de seguimento clínico e experimentais com o objetivo de explicar o prognóstico e as relações fisiopatológicas entre o tiol e as AVs em pacientes com SCA. Em terceiro lugar, outros parâmetros oxidativos como estado antioxidante total, estado oxidante total, paraoxonase e outros parâmetros de inflamação como PCR e fibrinogênio não foram avaliados em nosso estudo devido ao desenho do estudo. Por último, o efeito da revascularização nos parâmetros oxidativos não foi avaliado devido ao desenho do estudo. Novos estudos envolvendo mais pacientes com características clínicas mais homogêneas podem produzir resultados mais generalizantes e consistentes.

## Conclusão

Em conclusão, os parâmetros do tiol plasmático, que são marcadores de status oxidativo baratos e detectados rapidamente no sangue, podem ser usados para identificar pacientes com SCA com alto risco de desenvolvimento de AV intra-hospitalar. A correlação entre os níveis de troponina e tiol pode sugerir que o tiol pode ser um marcador importante para o diagnóstico e prognóstico da SCA com a ajuda de estudos futuros.
