# Supplementary material for: Associação entre Parâmetros Plasmático de Tiol e Níveis de Troponina em Pacientes com Síndrome Coronariana Aguda e Predição de Arritmia Ventricular Hospitalar
Source: Arq Bras Cardiol. 2021 Jul 7;117(3):465–73. [Article in Portuguese] doi: 10.36660/abc.20190672 (PMC8462948; doi:10.36660/abc.20190672)
Supplement: Translation

# Translation: Association between Plasma Thiol Parameters and Troponin Levels in Patients with Acute Coronary Syndrome and Prediction of In-Hospital Ventricular Arrhythmia

**Authors:** Mehmet Erdoğan, Selcuk Ozturk, Elçin Özdemir Tutar, Esma Arslan, Muhammet Cihat Çelik, Serdal Baştuğ, Salim Neşelioğlu

**Affiliations:** 1 Turquia Ministry of Health Ankara City Hospital-Cardiology, Ankara - Turquia; 2 Turquia Yozgat Bozok University Faculty of medicine-Cardiology, Yozgat - Turquia; 3 Turquia Yildirim Beyazit University Faculty of Medicine-Biochemistry, Ankara - Turquia; 4 Turquia Yildirim Beyazit University Faculty of Medicine-Biochemistry, Ankara - Turquia

**Keywords:** Atherosclerosis, Coronary Artery Disease, Coronary Acute, Syndrome, Oxidative Stress, Arrhythmias, Cardiacs, Acetyl- CoA-Acyltransferase

## Abstract

**Background:**

Ventricular arrhythmias (VAs) are the main cause of in-hospital mortality and morbidity in acute coronary syndrome (ACS) patients and its relationship with thiol is not known.

**Objective:**

To investigate the relationship between plasma thiol levels and troponin levels in patients with ACS and to estimate in-hospital VA development during hospital stay.

**Method:**

The study included 231 consecutive ST-segment elevation ACS (STE-ACS) and non-ST-segment elevation ACS (NSTE-ACS) patients. After application of exclusion criteria, 191 patients were included in the statistical analysis. Patients were classified into two groups: STE-ACS group (n=94) and NSTE-ACS group (n=97). Plasma thiol, disulphide and troponin levels were measured and troponin-to-native thiol ratio (TNTR) was calculated. A two-sided p value of less than 0.05 was considered to be statistically significant.

**Results:**

Plasma native thiol, total thiol, disulphide and their ratios were similar between the groups. TNTR was significantly higher in the STE-ACS group compared to the NSTE-ACS group. Troponin and thiol levels correlated negatively and significantly. Native thiol was found to be an independent predictor of VA development in STE-ACS patients and in all ACS patients. TNTR was found to be an independent predictor of VA development in NSTE-ACS patients and in all ACS patients.

**Conclusion:**

Plasma thiol levels can be used to identify ACS patients at high risk for in-hospital VA development. Correlation between troponin and thiol levels may suggest that thiols may be an important marker for diagnosis and prognosis of ACS with the help of future studies.

## Introduction

Atherosclerosis is a multifactorial disease caused by endothelial damage in people with risk factors such as dyslipidemia, diabetes, smoking and genetic predisposition. Acute coronary syndrome (ACS), which is a result of this condition, includes patients with persistent ST-segment elevation (STE-ACS) and patients without persistent ST-segment elevation (NSTE-ACS) detected by electrocardiography (ECG).^1^ STE is mostly associated with total acute coronary occlusion, whereas NSTE-ACS is mostly related with sensitive atherosclerotic plaque and subtotal thrombosis.

Over the last ten years, many studies have demonstrated the role of overproduction of reactive oxygen species (ROS) and resulting oxidative stress in the pathogenesis of atherosclerosis.^2^ Indeed, the metabolic process of ROS plays significant roles in the physiological functions of cells and this process is counterbalanced by the antioxidative system of the body. Oxidation of low-density lipoprotein (LDL) particles consist in the preliminary and principal phase of atherosclerosis. In addition, ROS formation contributes to apoptosis, inflammation and cell proliferation processes.^3^

Thiol and disulphide homeostasis status has a critical role in antioxidant protection, detoxification, apoptosis and regulation of enzymatic activity.^4^ Recent studies have shown that abnormal plasma thiol level and disulphide balance are associated with various cardiovascular diseases such as stable angina pectoris,^5^ acute myocardial infarction (MI),^6^ cardiac syndrome X,^7^ coronary slow flow,^8^ primary hypertension^9^ and diabetes mellitus (DM).^10^ It is known that ventricular arrhythmias (VAs), which may be due to myocardial necrosis, autonomic and electrolyte imbalance, acidosis and reperfusion injury, are the main causes of in-hospital mortality and morbidity in ACS patients.^11^ However, there is no study in the literature regarding the relationship between oxidative process, myocardial necrosis, and in-hospital VA development in ACS patients. In this context, we aimed to investigate the relationship between plasma thiol and troponin levels in patients with ACS and its association with VA development during in-hospital follow-up.

## Methods

This observational cross-sectional study was conducted from February to May 2018 and included 231 consecutive patients from our cardiology clinic. Patients hospitalized in the coronary intensive care unit diagnosed with ACS and who underwent coronary angiography were included in the study in a prospective manner. Patients with active infectious or inflammatory diseases, malignancy, hematological disorders, severe kidney disease, severe liver disease, rheumatic disease, severe valvular disease, patients taking antioxidants and/or under vitamin replacement therapy and patients in cardiogenic shock were excluded from the study. In addition, patients with insufficient clinical data were also excluded. The study protocol was approved by the local ethics committee (No: 39/Date: 21.02.2018) and was conducted in accordance with the ethical principles of the Declaration of Helsinki. Informed consent was obtained from each patient.

After the application of exclusion criteria, 191 patients were included in the statistical analysis. Patients were classified into two groups: STE-ACS group (n=94) and NSTE-ACS group (n=97). Diagnosis of ACS was made according to the current guidelines using history taking, ECG, imaging methods and troponin levels. Baseline clinical parameters of patients were evaluated. In-hospital mortality, VA occurrence and length of hospital stay were also evaluated and recorded. Diagnosis of STE-ACS included patients with acute onset chest pain and persistent ST-segment elevation on ECG.^12^ Diagnosis of NSTE-ACS included patients with acute onset chest pain, no persistent ST-segment elevation on ECG and/or cardiac troponin levels greater than the upper limit of the normal range.^13^ Arterial hypertension was defined as patients with repeated blood pressure measurements ≥140/90 mmHg or previous diagnosis of hypertension with antihypertensive medications. DM was defined as fasting plasma glucose levels higher than 126 mg/dL in multiple measurements or glucose level over 200 mg/dL at any measurement or active use of antidiabetic drugs and/or insulin therapy. Smoking was defined as current smoking in the previous six months. Hypercholesterolemia was defined as baseline cholesterol level of >200 mg/dl and/or LDL cholesterol level of >130 mg/dl or previously diagnosed and treated hypercholesterolemia.

In-hospital VA was defined as sustained or non-sustained ventricular tachycardia (VT) and ventricular fibrillation (VF). Diagnosis was determined from baseline ECG and/or telemetry recordings according to the clinical status of patients during hospital stay. In addition, telemetry recordings were retrospectively reviewed on the computer for other VA attacks before discharge. Ventricular premature beats with more than three consecutive beats were defined as VT. VTs lasting longer than thirty seconds were defined as sustained, and shorter duration was defined as non-sustained VT.

The Thrombolysis in Myocardial Infarction (TIMI) risk score consisting of 8 items, which predicts all-cause mortality at 30 days, was calculated for patients with STE-ACS and TIMI risk score consisting of 7 items, which predicts all-cause mortality, new or recurrent MI, or severe recurrent ischemia requiring urgent revascularization at 14 days, was calculated for NSTE-ACS patients.^14^ Calculation of Global Registry of Acute Coronary Events (GRACE) risk score was performed in all patients to estimate the probability of in-hospital death.^15^

Transthoracic echocardiography was performed using 3S-RS 1.5-3.6 MHz phased array transducer with General Electric Vivid 7 (GEMS Ultrasound, Israel) ultrasound device in the first 48 hours of hospitalization. Left ventricular ejection fraction (LVEF) was calculated from the apical 2 and 4 chamber images using the Modified Simpson’s method. Coronary angiography was performed using the Siemens Axiom Sensis XP device by cannulization of the femoral artery applying the standard Judkins technique. In addition to acetylsalicylic acid, all patients received dual antiplatelet therapy with clopidogrel, prasugrel or ticagrelor according to their clinical status.

Venous blood sample measurements of patients were taken upon admission. Routine biochemistry studies were performed on Hitachi 747 autoanalyzer. High-density lipoprotein (HDL) cholesterol was measured after dextran sulphate magnesium precipitation. LDL-cholesterol was calculated using the Friedewald method. Cholesterol, fasting triglycerides, HDL-cholesterol plasma concentrations were measured using the enzymatic chemical cleaning method using Cobas 6000 (Roche Diagnostics GmbH, Mannheim, Germany). Serum troponin I levels were quantitatively measured on the Elecsys 2010 analyzer (Roche Diagnostics, Basel, Switzerland). Serum levels of cardiac enzymes (CK, CK-MB and troponin I) were measured on admission and repeated every day. Plasma thiol and disulphide levels were measured according to the method developed by Erel et al. immediately upon admission. Blood samples were taken into tubes containing ethylenediamine tetraacetic acid (EDTA) to determine thiol and disulphide levels. Briefly, the degradable disulphide bonds were reduced to form free functional thiol groups. Subsequently, unused reductive sodium borohydride was used and extracted with formaldehyde and then all of the native and reduced thiol groups were determined after reaction with 5,5-dithiobis-(2-nitrobenzoic) acid. Dynamic disulphide levels were calculated by half of the difference between total and native thiol. After determination of native thiol and disulphide levels, native thiol-to-disulphide ratio was calculated. Using this method, dynamic thiol-to-disulphide ratio homeostasis was determined more easily and inexpensively in approximately ten minutes.^16^

## Statistical analysis

All analyses were carried out using IBM SPSS Statistics for Macintosh, Version 24.0 (IBM Corp., Armonk, New York, USA). One-sample Kolmogorov-Smirnov test was used to evaluate the distribution of numerical variables. According to the results of this test, independent two samples T-test was applied to the numerical data which conforms to the normal distribution and the results were entered as mean and standard deviation. On the other hand, Mann-Whitney U test was used for skewed distributed variables. Considering the results of this test, the median and interquartile range values were used. Chi-square test was used for categorical variables. Fisher’s exact test was applied in cases where the chi-square test could not be applied. For correlation analyses, Pearson’s correlation analysis was preferred for data with normal distribution. Otherwise, Spearman’s correlation analysis was preferred. Independent predictors of in-hospital VA were determined using logistic regression analysis. Variables that might have a clinical relationship with in-hospital VA such as LDL, age, LVEF, serum potassium and magnesium levels, neutrophil-lymphocyte ratio (NLR), admission time, troponin-to-native thiol ratio (TNTR), and troponin and native thiol were included in the logistic regression analysis. To avoid multicollinearity, we conducted multivariate analysis using three models separately. Each multivariate model included only one marker (TNTR, troponin, native thiol). Logistic regression analysis was performed separately for all groups with STE-ACS, NSTE-ACS and all ACS patients. Receiver operating characteristic (ROC) curve analysis was used to determine the cut-off values (Youden index^17^ ) for TNTR sensitivity and specificity in predicting VA. A two-sided *p* value of less than 0.05 was considered to be statistically significant.

## Results

Demographic characteristics, laboratory parameters and clinical features of the patient groups are shown in Table 1 . There was no difference between the groups in terms of age, gender, body mass index and atrial fibrillation. Percentage of hypertension, DM and admission time was higher in the NSTE-ACS group, whereas percentage of smokers was higher in the STE-ACS group. Neutrophil counts, platelet counts and NLR were significantly higher in the STE-ACS group compared to the NSTE-ACS group. Lymphocyte counts, hemoglobin, albumin, serum electrolytes including potassium, calcium, and magnesium, renal and liver function tests, and lipid parameters, except triglycerides, were comparable between the groups. Peak cardiac enzyme values including CK-MB and troponin were significantly higher in the STE-ACS group than in the NSTE-ACS group, whereas LVEF was lower in the STE-ACS group. Plasma native thiol, total thiol, disulphide, disulphide-to-native thiol ratio, disulphide-to-total thiol ratio, and native thiol-to-total thiol ratio was similar between the groups. However, TNTR was significantly higher in the STE-ACS group compared to the NSTE-ACS group. In addition, the GRACE score was significantly higher in the STE-ACS group compared to the NSTE-ACS group. A total of 23 patients developed in-hospital VA. Of the patients who developed in-hospital VA, 14 patients had sustained VA while 9 patients had non-sustained VA. There was no difference between the groups in terms of VA occurrence, length of hospital stay, and in-hospital mortality.

**Table 1:** Demographic characteristics and laboratory findings of the study population

Variables	STE-ACS (n=94)	NSTE-ACS (n=97)	p value
Age, years	58.7±11.1	62.1±13.7	0.06
Gender (male), n (%)	80 (85)	71 (73)	0.05
BMI (kg/m^2^)	26.9±6.9	28.5±5.6	0.14
Hypertension, n (%)	39 (41)	61 (62)	0.003*
Diabetes mellitus, n (%)	22 (23)	43 (44)	0.02*
Smoking, n (%)	65 (69)	44 (45)	0.001*
Atrial fibrillation, n (%)	0 (0)	8 (8)	0.07
Admission time, hours	2 (1–5)	28 (7–72)	<0.001*
Neutrophil count (K/uL)	7650 (5500–11525)	6100 (4600–8150)	<0.001*
Lymphocyte count (K/uL)	1900 (1222–3025)	1900 (1525–2500)	0.81
NLR	5.0 (2.0–7.9)	2.7 (2.1–4.5)	0.02*
Platelet count (K/uL)	252±79	231±63	0.04*
Hemoglobin (g/dl)	14.4±1.7	13.7±1.8	0.08
Albumin (g/dl)	4.1±0.4	4.2±0.3	0.21
Total cholesterol (mg/dl)	182±42	194±53	0.07
HDL (mg/dl)	37 (31–45)	39 (33–47)	0.06
LDL (mg/dl)	119±33.5	116±36.5	0.57
Triglycerides (mg/dl)	97.5 (60–154)	130 (91–189)	<0.001*
Potassium (mmol/L)	4.3±0.4	4.4±0.5	0.14
Creatinine (mg/dl)	0.9 (0.7–1.0)	0.9 (0.7–1.1)	0.31
GFR (mL/min/m^2^)	90 (72–144)	86 (60–96)	0.07
Calcium (mg/dl)	9.2±0.6	9.3±0.5	0.17
Magnesium (mg/dl)	2.0 (1.9–2.2)	2.0 (1.9–2.2)	0.79
AST (U/L)	30.5 (20–52)	30 (21–39)	0.34
ALT (U/L)	24 (16–34)	21 (14–30)	0.13
CK–MB Mass (ng/mL)	100 (26–209)	13 (3–66)	<0.001*
Troponin I (μg/L)	2571 (736–4810)	365 (43–1394)	<0.001*
LVEF (%)	43.9±8.5	49±11.2	<0.001*
Troponin/native thiol	6.7 (1.9 -15.8)	1.0 (0.1 - 3.8)	<0.001*
Plasma native thiol (µmol/l)	359.8±78.4	366.6±68.5	0.52
Plasma total thiol (µmol/l)	401.3±81.7	407.6±74.4	0.57
Disulphide (µmol/l)	20.1±10.8	19.0±11.9	0.53
Disulphide/native thiol	0.04 (0.03–0.08)	0.05 (0.03–0.07)	0.49
Disulphide/total thiol	0.04 (0.03–0.07)	0.04 (0.02–0.06)	0.53
Native thiol/total thiol	0.90 (0.86–0.94)	0.91 (0.87–0.95)	0.16
TIMI risk score	2 (1–4)	4 (3–5)	-
GRACE score	151.7±26.6	133.7±35.1	<0.001*
Ventricular arrhythmia, n (%)	13 (13.8)	10 (10.3)	0.45
Length of hospital stay, days	4 (3–5)	4 (3–5)	0.26
Mortality, n (%)	3 (3)	1 (1)	0.36

ALT: alanine transaminase; AST: aspartate transaminase; BMI: body mass index; CK-MB: creatine kinase-MB; GFR: glomerular filtration rate; HDL: high-density lipoprotein; IQR: interquartile range; LDL: low-density lipoprotein; LVEF: left ventricle ejection fraction; NLR: neutrophil/lymphocyte ratio; NSTE-ACS: Non-ST-segment elevation acute coronary syndrome; SD: standard deviation; STE: ST-segment elevation. Parameters were expressed as mean±SD and median [IQR]. * p<0.05 was considered significant for statistical analyses.

Correlation analysis of multiple variables with native thiol, total thiol and TNTR in the whole study population are presented in Table 2 . Native thiol levels were significantly and negatively correlated with age, GRACE score, NLR, hospital stay and troponin ( Figure 1A ), whereas significantly and positively correlated with GFR, LVEF and albumin. Total thiol levels were significantly and negatively correlated with age, GRACE score, hospital stay and troponin ( Figure 1B ), whereas significantly and positively correlated with GFR, LVEF and albumin. TNTR levels were inversely and significantly correlated with LVEF, and positively and significantly correlated with GRACE score, NLR, CKMB and hospital stay. Correlation coefficient was weak or moderate for all significant variables included in the analysis.

**Table 2:** – Plasma native thiol, total thiol and troponin/native thiol index parameters of acute coronary syndrome patients and their correlation with variables

Variables	Native Thiol		Total Thiol		Troponin-to-Native Thiol Ratio	

	r	p	r	p	r	p
Age	-0.25	<0.001*	-0.26	<0.001*	+0.06	0.34
GFR	+0.33	<0.001*	+0.33	<0.001*	-0.13	0.06
Grace score	-0.36	<0.001*	-0.35	<0.001*	+0.40	<0.001*
LVEF (%)	+0.19	0.009*	+0.18	0.01*	-0.42	<0.001*
NLR	-0.14	0.04*	-0.14	0.054	+0.34	<0.001*
Albumin	+0.39	<0.001*	+0.41	<0.001*	-0.12	0.07
Troponin	-0.27	<0.001*	-0.28	<0.001*	-	-
CK-MB Mass	-0.11	0.12	-0.10	0.15	+0.63	<0.001*
Hospital stay	-0.22	0.002*	-0.16	0.02*	+0.23	0.001*
LDL	+0.10	0.14	+0.13	0.06	+0.04	0.57

CK-MB: creatine kinase-MB; GFR: glomerular filtration rate; LDL: low-density lipoprotein; LVEF: left ventricular ejection fraction; NLR: neutrophil-lymphocyte ratio. *p<0.05 was considered significant for statistical analyses.

**Figure 1:** – *Relationship between troponin and native and total thiol levels.*

Logistic regression analysis of in-hospital VA predictors for STE-ACS patients, NSTE-ACS patients and all ACS patients are shown in Table 3 . Native thiol was found to be an independent predictor of VA development in STE patients, whereas TNTR was found to be an independent predictor of VA development in NSTE-ACS patients. When it comes to the whole ACS patient population, TNTR and native thiol were found to be independent predictors of VA development. In all ACS patients, the area under the curve for TNTR was 0.783 and the TNTR cut-off value (6.13) was associated with 78% sensitivity and 72% specificity to predict in-hospital VA as shown in Figure 2 .

**Table 3:** – Independent predictors of ventricular arrhythmia by logistic regression analysis

Variables	OR	95%CI		p value

		Lower	Upper	
**Ventricular arrhythmia/STE**				
LDL	1.021	0.997	1.045	0.08
Age	1.026	0.965	1.091	0.40
LVEF (%)	1.044	0.943	1.155	0.40
Potassium	0.849	0.214	3.366	0.81
Magnesium	1.072	0.121	9.518	0.95
NLR	1.142	0.997	1.308	0.05
Admission time	1.016	0.994	1.038	0.15
Troponin/native thiol	1.030	0.981	1.081	0.23
Troponin	1.000	1.000	1.000	0.64
Native thiol	0.962	0.937	0.986	0.03*
**Nagelkerke R** ^**2**^ **= 0.257, p: 0.07**				
**Ventricular arrhythmia/NSTE-ACS**				
LDL	0.970	0.940	1.001	0.05
Age	1.086	0.985	1.196	0.09
LVEF (%)	1.034	0.917	1.165	0.58
Potassium	1.278	0.218	7.488	0.78
Magnesium	0.105	0.001	11.00	0.34
NLR	1.102	0.919	1.321	0.29
Admission time	1.003	0.988	1.019	0.68
Troponin/native thiol	1.263	1.061	1.504	0.009*
Troponin	1.001	1.000	1.001	0.05
Native thiol	0.969	0.947	0.992	0.08
**Nagelkerke R** ^**2**^ **= 0.592, p<0.001**				
**Ventricular arrhythmia/All patients**				
LDL	1.001	0.987	1.015	0.90
Age	1.040	0.998	1.084	0.06
LVEF (%)	0.994	0.940	1.051	0.82
Potassium	0.867	0.346	2.169	0.76
Magnesium	0.681	0.096	4.845	0.70
NLR	1.087	1.000	1.182	0.05
Admission time	1.002	0.995	1.008	0.57
Troponin/native thiol	1.059	1.022	1.098	0.002*
Troponin	1.000	1.000	1.000	0.10
Native thiol	0.976	0.966	0.986	<0.001*
**Nagelkerke R2 =0.240, p: 0.001**				

Multivariate logistic regression analysis. The regression model included age, low-density lipoprotein (LDL), left ventricular ejection fraction (LVEF, %), serum potassium, serum magnesium, neutrophil-lymphocyte ratio (NLR) and hospital admission time as possible independent variables. 95% CI: 95% confidence interval; OR: odds ratio. * p<0.05 was considered significant for statistical analyses.

**Figure 2:** – *Receiver operating characteristic (ROC) curve analyses for prediction of ventricular arrhythmia development in all patients.*

## Discussion

The findings of this study demonstrated that there was a negative correlation between troponin and plasma thiol levels in ACS patients. Furthermore, especially in NSTE-ACS patients and in the whole ACS patient population, TNTR was shown to be a strong and independent marker for the prediction of in-hospital VA. To the best of our knowledge, this is the first study in the literature demonstrating the relationship between thiol and in-hospital VA development in patients with ACS.

One of the main pathophysiological mechanisms in atherosclerosis is increased oxidative stress with inflammation.^18^ Serum thiol level, which is an indicator of oxidative stress, can be manually detected by fluorescent capillary electrophoresis and/or luminescent systems. However, these systems are expensive, time consuming and difficult to apply. A colorimetric method developed by Erel et al. in 2014 has led to the automatic determination of thiol levels. Additionally, this new method is more accurate, faster and cheaper compared to previous methods.^16^ On the other hand, NLR, which is an easily accessible and inexpensive inflammatory marker, is a parameter that demonstrates systemic inflammatory response in various cardiovascular and non-cardiovascular conditions.^1^ In a study performed by Altiparmak et al.,^7^ total serum and native thiol levels were found to be lower in patients with cardiac syndrome X and total serum and native thiol levels were negatively correlated with NLR.^7^ In our study, NLR levels were significantly higher in the STE-ACS patient group compared to the NSTE-ACS patient group and there was a negative correlation between NLR and thiol levels in the whole ACS population. In the light of these findings, it is reasonable to speculate that plasma thiol, a marker of antioxidant protection in the human body, is negatively associated with increased inflammation in ACS patients.

Oxidative stress initiates the development of atherosclerosis through lipid peroxidation and formation of free radicals at first stages, and through vascular inflammation at advanced stages.^19^ Instability of atherosclerotic plaque is triggered by increased oxidative stress and reactive oxygen radicals while antioxidant capacity decreases during this process. Subsequently, the balance of oxidative and antioxidant system, which is an important regulator of cellular protection, detoxification, apoptosis and enzymatic activities, begins to deteriorate.^20^ A previous study showed that levels of oxidative stress markers increase after MI and reperfusion.^21^ In our study, there was a negative and statistically significant correlation between plasma thiol levels and age and troponin, whereas there was a positive and statistically significant correlation between thiol levels and LVEF. These results were consistent with a previous study published by Kundi et al..^6^ Sivri et al. demonstrated lower plasma total thiol and native thiol levels in NSTE-ACS patients compared to the control group patients. In addition, they demonstrated an inverse relationship between the GRACE score and total plasma thiol and native thiol levels.^22^ According to our study, which included STE-ACS and NSTE-ACS patients, a significant negative correlation was observed between the GRACE score and thiol parameters. Correlation of thiol with these strong diagnostic and prognostic markers such as troponin and the GRACE score might underline that it may be an important marker for diagnosis and prognosis of ACS with the help of future studies.

More than 80% of sudden cardiac deaths are due to atherosclerotic coronary artery disease (CAD), and the most common cause is VAs due to ischemia resulting from CAD and ACS.^23^ Therefore, early prediction, diagnosis and effective treatment of VAs is very important. During oxidative stress, sensitivity of elderly hearts to VF increases. In one study, oxidative stress induced by hydrogen peroxide was shown to be associated with increased early afterdepolarizations and triggered activity in ventricular myocytes.^24^ Although the myocardium has a strong defense mechanism, it is susceptible to oxidative stress because of its high workload and oxygen need. As a result of ischemia, there is a decrease in antioxidant levels such as mitochondrial superoxide dismutase and intracellular glutathione against free oxygen radicals. In addition, the production of free oxygen radicals increases in mitochondria and leukocytes, and production of oxygen metabolites increases during the reperfusion period, which can be more toxic by re-introduction of oxygen. Oxidative stress firstly causes oxidation of thiol groups and this leads to a reversible damage at the initial stage. At later stages, this process leads to accelerated necrosis and predisposition to arrhythmias. In the early period, it may induce myocardial membrane electrical imbalance, alterations in the permeability of ion channels and cause induction of arrhythmogenic changes in the potential pattern of ventricular action.^21^ An increase in the duration of action potential was observed after exposure to free oxygen radicals followed by the appearance of early and after depolarizations.^25 , 26^ In the light of the previously published reports, we have hypothesized that thiol levels may predict VA development in ACS patients and found that TNTR can be used to predict VA development in NSTE-ACS patients but not in STE-ACS patients. This disagreement may originate in the pathophysiological and clinical diversities between STE-ACS and NSTE-ACS. STE-ACS is a result of total occlusion of the vessel with fibrin-rich clot, whereas NSTE-ACS is a result of subtotal occlusion of the vessel with platelet-rich clot. Additionally, hospital admission time is shorter in STE-ACS patients and, consequently, revascularization time is faster compared to NSTE-ACS patients. Therefore, it is reasonable to speculate that there is not sufficient time for oxidative process to progress to plasma at the cellular level in STE-ACS patients. However, due to the fact that revascularization time is longer in NSTE-ACS patients, progression of oxidative stress from cardiomyocytes to plasma can be more pronounced. In addition, NSTE-ACS patients are older than STE-ACS patients and suffer from comorbidities such as DM and/or hypertension more often compared to STE-ACS patients as shown in our study group. These comorbidities might have affected the oxidative status in NSTE-ACS patients.

Our study has several limitations. Firstly, the number of samples is relatively small and this is a single-center study. Secondly, this is an observational study and it should be supported by further clinical follow-up and experimental studies aiming to explain prognosis and pathophysiological relationships between thiol and VAs in ACS patients. Thirdly, other oxidative parameters such as total antioxidant status, total oxidant status, paraoxonase and other inflammation parameters such as CRP and fibrinogen were not evaluated in our study due to the study design. Lastly, the effect of revascularization on oxidative parameters has not been evaluated due to the study design. Further studies involving more patients with more homogenous clinical characteristics may produce more generalizing and consistent results.

## Conclusion

In conclusion, plasma thiol parameters, which are inexpensive oxidative status markers detected rapidly in blood, can be used to identify high risk for in-hospital VA development in ACS patients. Correlation between troponin and thiol levels may suggest that thiol may be an important marker for diagnosis and prognosis of ACS with the help of future studies.
